# Highly multiplexed imaging of single cells using a high-throughput cyclic immunofluorescence method

**DOI:** 10.1038/ncomms9390

**Published:** 2015-09-24

**Authors:** Jia-Ren Lin, Mohammad Fallahi-Sichani, Peter K. Sorger

**Affiliations:** 1 HMS LINCS Center & Laboratory of Systems Pharmacology, Harvard Medical School, Boston, Massachusetts 02115 USA; 2 Department of Systems Biology Harvard Medical School 200 Longwood Avenue, Boston, Massachusetts 02115 USA

## Abstract

Single-cell analysis reveals aspects of cellular physiology not evident from population-based studies, particularly in the case of highly multiplexed methods such as mass cytometry (CyTOF) able to correlate the levels of multiple signalling, differentiation and cell fate markers. Immunofluorescence (IF) microscopy adds information on cell morphology and the microenvironment that are not obtained using flow-based techniques, but the multiplicity of conventional IF is limited. This has motivated development of imaging methods that require specialized instrumentation, exotic reagents or proprietary protocols that are difficult to reproduce in most laboratories. Here we report a public-domain method for achieving high multiplicity single-cell IF using cyclic immunofluorescence (CycIF), a simple and versatile procedure in which four-colour staining alternates with chemical inactivation of fluorophores to progressively build a multichannel image. Because CycIF uses standard reagents and instrumentation and is no more expensive than conventional IF, it is suitable for high-throughput assays and screening applications.

## Abstract (web summary)

Multiplexed single cell measurements provide insight into connections between cell state and phenotype. Here Lin *et al.* present CycIF, a high throughput, public domain immunofluorescence method for multiplexed single-cell analysis of adherent cells following live-cell imaging.

## Graphical abstract

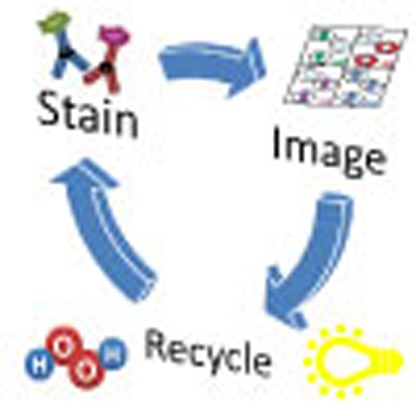

Increasing the multiplicity of single-cell measurement (the number of independent measurements performed on each cell) has the potential to reveal interdependencies among differentiation status, signal-transduction state, local environment and phenotype that are not evident when the same measurements are made independently or performed at the population level^1^,^2^,^3^,^4^. Multiplex data on cell-to-cell fluctuations can also be used to characterize signalling pathways in new ways, as illustrated by methods such as Wanderlust and viSNE^5^,^6^. Relative to flow-based methods, in which >30 channels can be recorded per cell^3^, immunofluorescence (IF) is typically limited to 4–6 channels. A compensatory advantage of imaging is that it reports on spatial features such as cell shape and protein localization and can be applied to living cells using dyes and genetically encoded reporters. Many technologies have been developed to increase the multiplicity of IF microscopy, including infrared-shifted fluorophores, quantum dots and bar coding^7^,^8^,^9^,^10^, but these techniques usually require special reagents or instrumentation, such as hyperspectral microscopes, which are not widely available and have their own limitations. With one channel used for image segmentation and registration, we find that 3–4 data channels is a typical limit for robust, high-throughput IF imaging in 96- and 384-well plates, particularly when signals are relatively weak or fluorescent proteins (FPs) are being imaged (most FPs have small Stokes shifts). Promising methods for overcoming this limit using rounds of antibody staining followed by stripping and restaining have been described^11^,^12^, but these methods (i) are proprietary and expensive (currently available only as a fee-for-service), (ii) have not been demonstrated for dyes and FP fusions and (iii) are not integrated into typical workflows for live-cell or high-throughout imaging.

In this paper we describe a robust approach to highly multiplexed imaging that overcomes the complexity of existing approaches by building a multiplex imaging four to six channels at a time. This involves repeated rounds of immunofluorescence staining and fluorophore inactivation. Cyclic immunofluorescence (CycIF) exploits chemistry in the public domain, uses commercially available antibodies, can be performed on conventional microscopes, and is sufficiently inexpensive for routine analysis of samples in 96-/384-well plates. The concepts underlying CycIF are quite old and therefore difficult to credit to their originators. The first procedures for increasing the multiplicity of cell and tissue staining by cycling the sample involved heat and exposure to acid^13^,^14^,^15^. Chemical methods for blocking the first of a series of staining reactions have also been described^16^,^17^, but the most obvious antecedent to CycIF involves removing sets of primary and secondary antibodies using a stripping process (first demonstrated for immunoblots^18^ that involves low pH, heat, salt, detergents and/or denaturing agents^19^) followed by another round of staining.

Oxidation of dyes with hydrogen peroxide, which can be catalysed using either acidic or basic conditions, is a well-known procedure for changing the fluorescent properties of dyes and fluorescent proteins (a white paper on this topic can be found at http://www.biotek.com/resources/articles/reactive-oxygen-species.html). In recent years peroxide-dye reactions have been used as means to probe reactive oxygen species (ROS) in fixed and living cells^20^ (for example, using genetically encoded reporters^21^). We have not identified a citation for acid and base catalysed-oxidation of Alexa Fluor Dyes (which are made by Life Technologies) but this seems to us a straightforward extension of existing chemistry. However, in a series of proprietary and patented modifications, Gerdes *et al.*^11^ describe means to selectively bleaching one dye while leaving a control dye relatively untouched. The simple procedure we describe nonetheless appears to be adequate for sequential assembly of a multichannel image. We also describe several routines for processing multichannel images, which is a field unto itself, but a wide variety of software methods should be equally good; http://loci.wisc.edu/software/home provides a good introduction.

## Results

### Conceptual overview of cyclic immunofluorescence

A typical 5-cycle CycIF procedure yields 16-channel images of slides or plates at a cost of ∼$2/sample (in a 384-well plate) and can be performed at the end of FP-based live-cell imaging. There is no theoretical limit to the number of CycIF cycles and channels. However, 8–16 channel images already present an analytical challenge and most of our work is therefore concentrated on validating different panels of antibodies for 4–5 cycle CycIF. In this paper we demonstrate the application of CycIF to drug response at a single-cell level and show that multiplex single-cell measurement reveals new aspects of drug sensitivity and resistance^22^,^23^; further refinement of the method will be reported at our web site at http://lincs.hms.harvard.edu/lin-NatCommun-2015.

**Figure 1:**
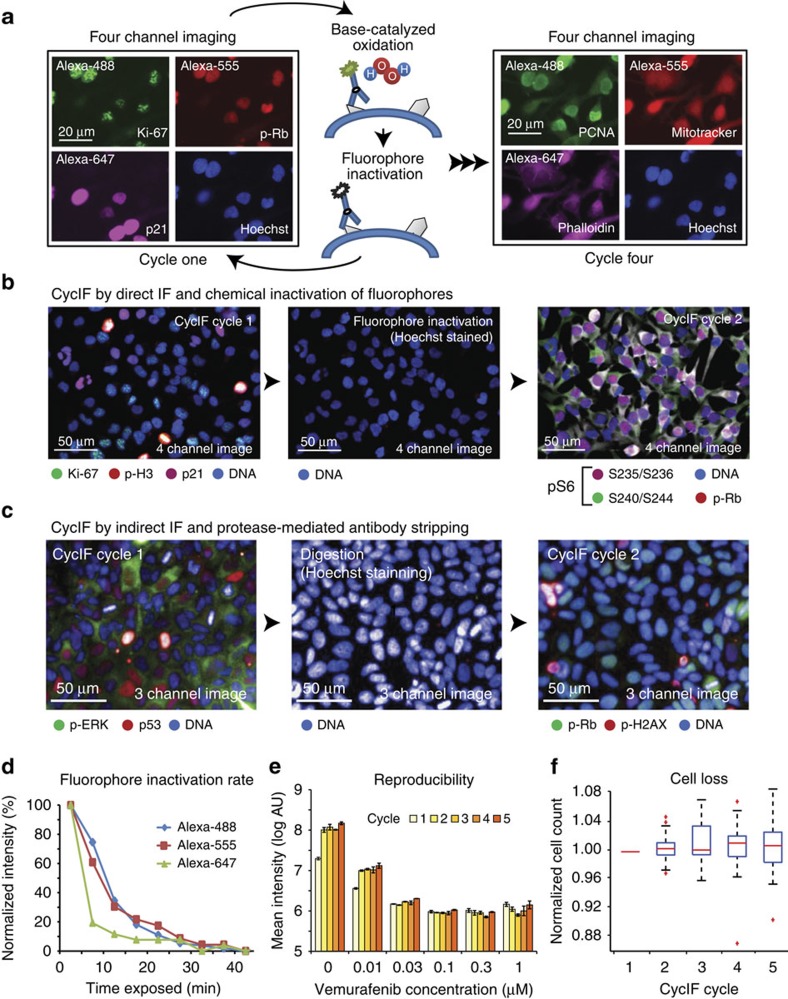
Multiplexed imaging of single-cell using Cyclic ImmunoFluorescence (CycIF). (**a**) An overview of the CycIF procedure. Four-colour staining alternates with fluorophore inactivation by oxidation to progressively build a multichannel image. (**b**) CycIF procedure using direct immunofluorescence (with fluorophore-conjugated antibodies) and chemical inactivation of fluorophores. COLO858 melanoma cells were fixed and stained using antibodies for Ki-67 (Alexa 488), p-Histone H3 (Alexa 555), p21 (Alexa 647) and Hoechst (left panel). Cells were exposed to fluorophore-inactivation by oxidation using hydrogen peroxide, high pH and light and then reimaged (middle panel) to confirm efficient bleaching. Cells were then stained with fluorophore-conjugated antibodies for p-S6^S240/244^ (Alexa 488), p-Rb^S807/811^ (Alexa 555), p-S6^S235/236^ (Alexa 647) and Hoechst. (**c**) CycIF procedure using indirect immunofluorescence and protease-mediated antibody stripping. MCF7 cells were fixed and stained using primary antibodies for p-ERK1/2^T202/Y204^ (rabbit), p53 (mouse), Alexa 488-conjugated anti-rabbit, and Alexa 647-conjugated anti-mouse secondary antibodies (left panel). Cells were digested with pepsin/papain mixture (see Methods for details) and reimaged (middle panel). Cells were restained using primary antibodies for p-Rb^S807/811^ (rabbit), p-Histone H2A.X^S139^ (mouse), Alexa 488-conjugated anti-rabbit, and Alexa 647-conjugated anti-mouse secondary antibodies (right panel). (**d**) Bleaching rate for Alexa 488, 555 or 647-conjugated antibodies following incubation in a base-hydrogen peroxide mixture. (**e**) Correlation of signal intensities after using the same antibodies in successive CycIF cycles. Five-cycle CycIF was applied to COLO858 cells treated with increasing doses of vemurafenib (error bars show the range of biological duplicates). Cells were stained with Alexa 488-conjugated p-ERK antibody, and p-ERK signal intensities from different CycIF rounds were quantified and compared. Cells (1,000–2,000) were imaged for each condition and well mean intensity values across duplicates were reported. Error bars indicate s.d. (**f**) Quantification of cell loss based on Hoechst staining (averaged across *n*=30 different wells) through cycles of CycIF. Cell numbers from each well after each CycIF cycle were normalized to the mean cell number derived from cycle 1 and presented in box-and-whisker plots with mean values (shown by red lines), interquartile ranges (shown as boxes) and whiskers (representing the 1st/99th percentiles).

CycIF has several variants (Supplementary Fig. 1), but the most generally useful involves three-channel IF using antibodies that are chemically coupled to one of three different Alexa dyes, while the fourth channel is used for Hoechst 33342 to visualize DNA and count cells. Fluorophores are then inactivated by oxidation using hydrogen peroxide^24^, high pH and light, followed by a wash step and a second round of staining and imaging (Fig. 1a,b, Supplementary Fig. 2). Indirect-IF can also be used, but since the primary antibodies remain bound to their antigens, the number of channels is limited to the number of different species (rabbit, rat, goat and so on) of primary antibody and thus the number of unique secondary antibodies. A second CycIF variant enables multiple rounds of indirect-IF by using proteases such as papain or pepsin to digest primary and secondary antibodies (Fig. 1c, Supplementary Fig. 3); this protocol is a variation on ‘epitope recovery’ techniques^25^. In a third CycIF variant, cells carrying FPs and other live-cell reporters are imaged and then bleached using low pH and hydrogen peroxide, followed by rounds of direct and indirect immunofluorescence. The use of a common dye in all cycles (Hoechst 33342 in the current work) makes it possible to assay for cell loss and register successive images at sub-pixel resolution (Fig. 1b; Hoechst is partially bleached by oxidation and we typically readd it at each cycle).

### Sensitivity and reproducibility of CycIF method

After each round of fluorophore oxidation we reimage cells to count them and to confirm that antibody-conjugated fluorophores have been fully inactivated. We find that Alexa 488, 555 and 647-bleach at different rates, but 30–45 min of incubation in a base-hydrogen peroxide mixture is sufficient to reduce fluorescence to prestaining levels (Fig. 1d, Supplementary Fig. 4). White light accelerates the reaction but is not essential. We find that, when the same antibody is used in successive CycIF cycles, signal intensity is highly correlated and morphology retained, as illustrated by p-ERK, p-S6^S240/244^, Ki-67 and PCNA in BRAF^V600E^ melanoma cells treated with the Raf-specific kinase inhibitor vemurafenib to create samples in which levels of ERK and S6 phosphorylation vary in a predictable manner (Fig. 1e, Supplementary Figs 5 and 6). The CycIF procedure does not measurably reduce immunogenicity as the number of cycles increases and in some cases signal-to-noise actually improves, presumably because samples become increasingly well blocked (although we do not have a quantitative metric of this improvement). Thus, it appears that, with the inclusion of appropriate controls, CycIF is as quantitative on a per-cell basis as conventional IF methods. Moreover, the fraction of cells lost through cycles of washing and imaging is low, approximately 2–5% in the first and second cycles, during which adherent cells detached in the NaOH-H_2_O_2_ mixture, and 0–2% per cycle subsequently (Fig. 1f). Low cell loss is partly a consequence of our use of plate washers for washing and staining steps (see Methods) and paraformaldehyde fixation; the use of methanol fixation alone results in higher levels of cell loss (although not all antibodies are compatible with paraformaldehyde). In the case of protease-mediated antibody inactivation (protocol variant 2), the mix of protease and digestion time must be optimized for each set of samples to balance antibody inactivation with retention of cell number and immunogenicity (in general, papain and pepsin work with most cell lines; Supplementary Fig. 3).

### Combining CycIF with live-cell imaging

**Figure 2:**
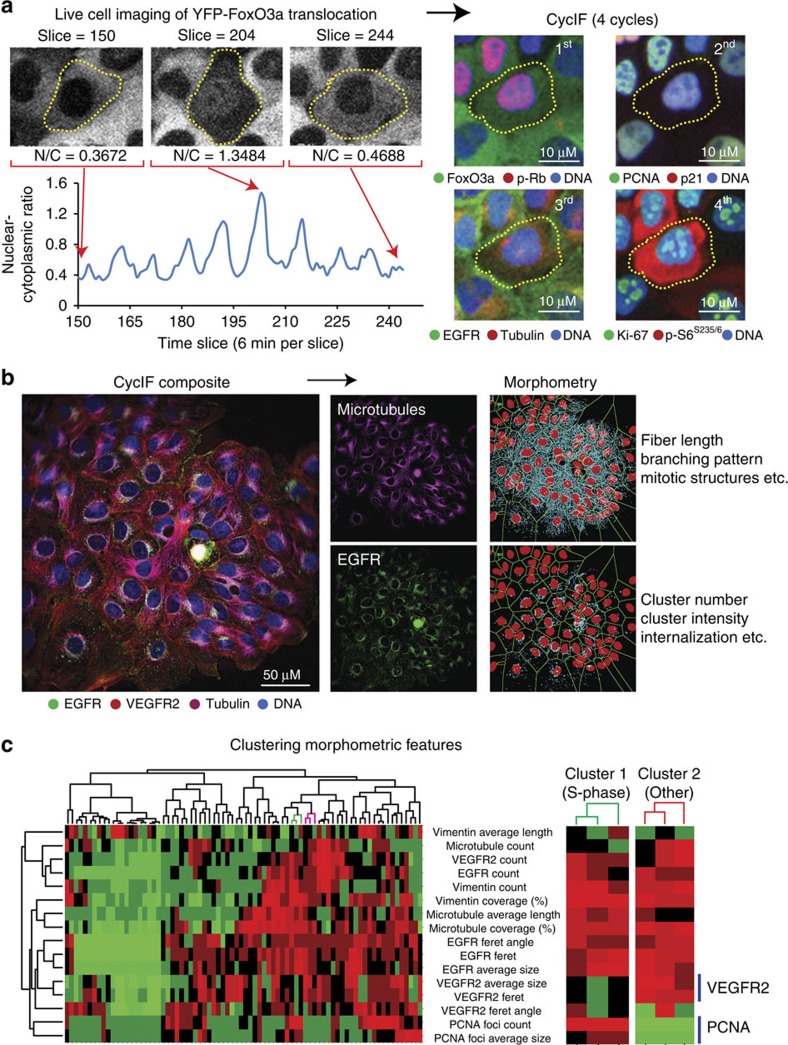
Compatibility of CycIF with live-cell imaging, analysis of subcellular localization of proteins and morphometric features. (**a**) Applying CycIF at the end of FP-based live-cell imaging. Nuclear translocation of FoxO3a in MCF10A cells (stably expressing YFP-FoxO3a and mCherry-NLS) was monitored for 24 h (left panel). Red arrows indicate FoxO3a nuclear to cytoplasmic (N/C) ratios at two snapshots. Cells were then fixed and subjected to four-cycle CycIF using fluorophore-conjugated antibodies p-Rb^S807/S811^ (cycle 1), p21 and PCNA (cycle 2), EGFR and β-tubulin (cycle 3) and Ki-67 and p-S6^S235/6^ (cycle 4) (right panel). (**b**) Morphometric analysis of CycIF-generated images of single cells. Three rounds of CycIF staining were performed as indicated in Supplementary Fig. S8. The raw intensity-based images of EGFR, VEGFR2, Tubulin, Vimentin and PCNA were then binarized and passed through different filters (sharpen, skeletonized and maximum, and so on) for extracting texture features (length, branches, enrichment and clusters). Two representative images/masks from Tubulin/microtubule and EGFR are shown here. Detailed methods and other images/masks could be found in Supplementary Information. (**c**) Single-cell clustering based on morphometric features. Sixteen different features were extracted from five different IF signals. The numerical values from features were used in hierarchical clustering of single cells (left panel). Two sub-clusters were displayed with distinct features generated from PCNA and VEGFR2 (right panel). The hierarchical clustering was performed in Matlab using Euclidean distance metrics and average linkage.

CycIF can be effectively combined with live-cell imaging. In the four-cycle, 9-channel experiment shown in Fig. 2a, we monitored nuclear translocation of FoxO3a in MCF10A cells stably expressing YFP-FoxO3a and mCherry-NLS for 24 h followed by fixation and four cycles of fluorophore inactivation and antibody staining. We used conjugated antibodies to measure cell cycle state (p-Rb^S807/S811^, p21, Ki-67 and PCNA; see Supplementary Tables 1 and 2 for the list of validated antibodies and abbreviations), signal transduction (EGFR and p-S6^S235/6^) and cell morphology (microtubules). In other experiments we also used dyes such as MitoTracker Red to label mitochondria and other organelles (Supplementary Fig. 7). The choice of which antibodies to combine at each step is made empirically, with the goal of assigning Alexa 647 to the weakest signals since the near-infrared has less autofluorescence. Relative to flow cytometry (or CyTOF), imaging adds information on cell morphology and protein translocation, which is particularly useful for studying the cytoskeleton, membrane receptor clustering and nuclear foci. In the images shown in Fig. 2b and Supplementary Fig. 8, we performed three rounds of CycIF and morphometric features then extracted from five channels using Bayesian classifiers and similar methods^26^. The dimensionality of such data is substantially higher than the number of unique antibodies or dyes and has the advantage, relative to flow cytometry, that features such as nuclear foci or receptor clusters can be scored and correlated (Fig. 2c). For example, VEGFR2 clustering appears to be negatively correlated with the number of PCNA foci, which is a marker of S-phase (Fig. 2c, left panels).

### Analysis of CycIF data

**Figure 3:**
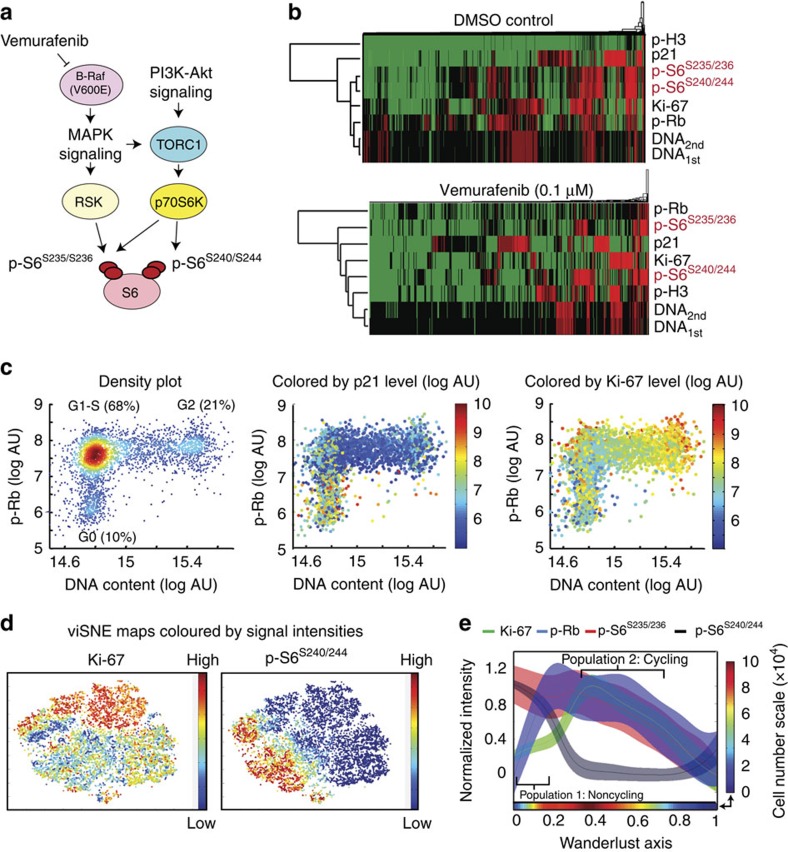
Multivariate single-cell analysis of BRAF^V600E^ COLO858 melanoma cells using CycIF. (**a**) Highly simplified schematic showing crosstalk between MAPK and PI3K/Akt/mTOR pathways at the level of S6 ribosomal protein phosphorylation. (**b**) Unsupervised clustering of single-cell 8-channel CycIF data in cells treated with DMSO or 0.1 μM vemurafenib for 24 h. The hierarchical clustering was performed in Matlab using Euclidean distance metrics and average linkage. (**c**) Scatter plots comparing three channels at a time (p-Rb, Hoechst, p21 and Ki-67) to determine the distribution of G0, G1/S and G2 cell cycle states. (**d**) viSNE-generated two-dimensional projections of the single-cell 8-channel CycIF data. Single-cell data are plotted in viSNE axes and coloured based on Ki-67 (left) and pS6^S240/244^ (right) signal intensities. (**e**) Wanderlust trace showing signal intensities for p-Rb^S807/811^, pS6^S235/236^, Ki-67 and pS6^S240/244^ across a five-point vemurafenib dose–response (0–1 μM) data set. The colour-box on the bottom represents the cell densities while the colour scale is showed on the side.

As a biologically relevant test of CycIF we imaged BRAF^V600E^ melanoma cells (Fig. 3, Supplementary Fig. 9), in which the therapeutically beneficial effects of Raf inhibition are opposed by counter-therapeutic upregulation of MAPK, Akt/mTOR and other compensatory progrowth pathways (so-called adaptive responses^27^). The level of ribosomal protein S6 phosphorylation is the best-validated biochemical marker of growth inhibition by vemurafenib since S6 lies downstream of both MAPK and Akt/mTOR signalling cascades^22^,^28^. The doubly phosphorylated sites pS6^S235/236^ and pS6^S240/244^ are jointly modified by p70 S6-Kinase 1, which is regulated by TORC1^29^; pS6^S235/236^ is also phosphorylated by the p90 ribosomal S6 kinase (RSK), which is regulated by the Raf/MEK/ERK cascade (Fig. 3a)^30^. Unsupervised clustering of single-cell eight-channel CycIF data show that pS6^S235/236^ and pS6^S240/244^ levels are strongly correlated at a single-cell level in dimethyl sulfoxide (DMSO)-treated cells (with Pearson’s correlation coefficient *R*^2^=0.90) but less so following exposure to 0.1 μM vemurafenib (*R*^2^=0.53) for 24 h (Fig. 3b) implying a breakdown in the relationship between the S6 regulatory processes. Analysis of high-dimensional data remains challenging but scatter plots enable three-way comparison. For example, by staining for p-Rb and Hoechst and either p21 or Ki-67 we can easily visualize the expected distribution of these markers through G0, G1/S and G2 cell cycle states (Fig. 3c). viSNE, an algorithm developed for visualizing CyTOF data^5^, generates two-dimensional projections that reveal the underlying correlation structure of complex data (Fig. 3d). Wanderlust, a trajectory detection algorithm also developed for CyTOF data^6^, maps high-dimensional single-cell data to a one-dimensional path on which cells are ordered based on their most probable placement along a continuum of transitional cell states (in this case, across a five-point vemurafenib dose–response data set; Fig. 3e). Combining these methods we see that, at intermediate doses of vemurafenib, pS6^S235/236^ and pS6^S240/244^ de-correlate and two separate sub-populations of cells emerge: (1) non-cycling Ki-67^Low^ cells that are p-S6^S235/236(High)^ and p-S6^S240/244(High)^, and (2) cycling Ki-67^High^ cells that are p-S6^S235/236(High)^ and p-S6^S240/244(Low)^. The latter population represents proliferating cells in which the ERK/RSK pathway is more active on S6 and probably plays a dominant role in proliferation. The former population appears to represent quiescent cells with high TORC1 activity that are also resistant to vemurafenib-induced apoptosis^22^,^28^. From these data we conclude that CycIF, combined with algorithms developed for flow cytometry and CyTOF, can exploit natural cell-to-cell fluctuations in protein levels and activities to uncover aspects of drug and ligand response that are obscured by population-average measurement^31^.

## Discussion

In summary, we report a robust public-domain method for highly multiplexed single-cell microscopy. The method involves variants for fluorophore-conjugated antibodies, indirect immunofluorescence and FP fusions and can be performed at a cost of a few dollars per well. We have demonstrated 15-channel imaging thus far, but because signals do not appreciably decay with more cycles, the multiplexing limit is presumably considerably higher. Moreover, the amount of information obtained at each cycle is higher than the channel number (or the equivalent information from flow cytometry) because each image is associated with multiple morphological features that can be automatically scored and analysed. CycIF can also be combined with live-cell imaging to exploit the advantages of both methods. From a computational perspective, CycIF data can be analysed using conventional imaging software or the algorithms recently developed for CyTOF data, but integrated analysis of multichannel intensity and morphometric data will require new tools. Future development of CycIF includes extending the number of cycles, validating more antibodies, applying the method to multi-cellular samples and tissues and improving analytical tools. We will report these and other updates at http://lincs.hms.harvard.edu/lin-NatCommun-2015/.

## Methods

### Immunostaining

Cells were fixed in 4% paraformaldehyde for 30 min at room temperature and washed three times with PBS, permeabilized in ice-cold methanol for 10 min at room temperature, rewashed with PBS and blocked in Odyssey blocking buffer (LI-COR) for 1 h at room temperature. Cells were incubated overnight at 4 °C with primary antibodies in blocking buffer. For staining with fluorophore-conjugated primary antibodies, cells were washed three times with PBS and stained with Hoechst 33342 (0.1 μg ml^−1^) for 15 min at room temperature. For primary antibodies that were not fluorophore-conjugated (that is, for indirect immunofluorescence), cells were washed three times with PBS, incubated with fluorophore-conjugated secondary antibodies in blocking buffer for 1 h at room temperature, washed with PBS and then stained with Hoechst 33342 for 15 min at room temperature. The list of validated antibodies and their sources are provided in Supplementary Table 1. Phalloidin-555 (catalog number P1951) was obtained from Sigma and MitoTracker-644 from Invitrogen (Cat. No. M22426).

### Chemical inactivation of fluorophores

Alexa 488/555/647 dyes were typically inactivated in 96-well plates although other formats are compatible with the method. After image acquisition, cells were washed three times with PBS (250 μl per well) using a Bio-Tek EL406 plate washer with final aspiration so as to empty each well. Hundred microlitres of a mixture of 3% H_2_O_2_ and 20 mM NaOH in PBS (137 mM NaCl, 2.7 mM KCl, 10 mM Na_2_HPO_4_ and 1.8 mM KH_2_PO_4_; pH≈9.5) were added to each well for 1 h at room temperature and continuously illuminated with an ordinary incandescent table lamp. To monitor for fluorophore inactivation, remaining fluorescence was measured on a microscope before removal of the oxidation solution. Cells were then washed three times with 250 μl PBS using a plate washer and reincubated with blocking buffer. After blocking, samples were subjected to the next round of staining, as described above. H_2_O_2_ was obtained as a 30% solution from Sigma (Cat. No. H1009), NaOH as pellets (Cat. No. S5881), and hydrochloric acid as a 37% (12 M) solution (Cat. No. 258148).

### Protease-mediated antibody stripping

For protease-based CycIF, cells imaged using indirect immunofluorescence were digested in 96-well format using pepsin, papain or trypsin. Conditions including the type of protease, concentration and digestion time required for maximal fluor-inactivation were balanced against cell loss, which increased with the extent of digestion. Conditions for each cell line were optimized independently (Supplementary Fig. 4). Stock solutions of pepsin (1% w/v in PBS, Sigma:P6887), papain (1% w/v in PBS, Sigma:P4762) and trypsin (0.05% w/v Invitrogen:25300-054) were diluted 1:100, 1:50, 1:25 in PBS and added to fixed and stained cells at room temperature. The progress of digestion was monitored using time-lapse imaging (6 min per frame) to determine remaining fluorescence levels and to monitor cell loss using the Hoechst channel. After antibody digestion, cells were gently washed twice with 300 μl PBS and then re-fixed in 4% paraformaldehyde for 20 min at room temperature. Cells were washed again with PBS and reblocked with Odyssey blocking buffer prior to starting the next cycle of immunofluorescence staining.

### Inactivation of fluorescent proteins

Inactivation of EGFP/EYFP and mCherry was performed in 96-well plates. Following live or fixed-cell image acquisition of cells with genetically encoded reporters, cells were washed three times with PBS (250 μl per well) using a Bio-Tek EL406 plate washer with final aspiration so as to empty each well. 100 μl of 3% H_2_O_2_ and 20 mM HCl in PBS (pH≈2.5) were added into each well for 1 h at room temperature with light illumination as described above. The progress of the reaction was monitored on a microscope before solution removal; when fluorescence intensity had fallen to background levels, cells were washed three times with 250 μl PBS using a plate washer, reincubated with blocking buffer and processed for the next cycle of staining.

### Antibodies

The specific animal sources, catalogue numbers and dilutions for fluorophore-conjugated antibodies from Cell Signaling Technology are listed in Supplementary Table 1. The source and dilution of unconjugated antibodies used in this study are as follows: p-Rb^S807/S811^ (rabbit mAb; Cell Signaling:#8516 1:1,000); p-cJun^S73^ (rabbit mAb; Cell Signaling:#3270; 1:500 ); c-Jun (rabbit mAb, Cell Signaling:#9165; 1:500); p53 (mouse mAb, Santa Cruz Biotech. sc-126, 1:200); γH2A.X (mouse mAb, Millipore: 05-636, 1:1,000); Goat-anti-Rabbit IgG, Alexa-488 conjugate (Invitrogen:A-11034, 1:2,000); Goat-anti-Mouse IgG, Alexa 647 conjugate (Invitrogen:A-21235, 1:2,000). In Supplementary Table 3, we listed all the antibodies we have already tested in CycIF protocol, including antibodies from several different vendors (Cell Signaling Technology, Abcam, Santa Cruz Technology and Biolegend). More updates will be available in the HMS-LINCS website (http://lincs.hms.harvard.edu/lin-NatCommun-2015/).

### Cell culture and reagents

Melanoma cell lines used in this study were obtained from the Massachusetts General Hospital Cancer Center. Primary sources of the cell lines are as follows: Colo858 (ECACC), C32 (ATCC), WM115 (ATCC), LOXIMV1 (DCTD Tumor Repository, National Cancer Institute), and WM1552C (ATCC). C32 and WM115 cell lines were grown in DMEM/F12 (Invitrogen) supplemented with 5% fetal bovine serum (FBS) and 1% sodium pyruvate (Invitrogen). COLO858, LOXIMVI and WM1552C cell lines were grown in RMPI 1640 (VWR) supplemented with 5% FBS and 1% sodium pyruvate. MCF7 and MCF10A cells were obtained from ATCC. MCF7 cells were grown in DMEM high glucose (Invitrogen) plus 10% FBS. MCF10A cells were maintained in DMEM/F12 (Invitrogen), containing 5% horse serum (Invitrogen), 20 ng/ml EGF (PeproTech), 0.5 mg ml^−1^ hydrocortisone (Sigma), 100 ng/ml cholera toxin (Sigma) and 10 μg ml^−1^ insulin (Sigma). We added penicillin (50 U ml^−1^) and streptomycin (50 μg ml^−1^) to all growth media. Cells were seeded at a density of 5,000 cells per well in 96-well plates (Corning) in full growth media for 24 h. Cells were then exposed to the indicated doses of vemurafenib or other compounds for 24 h using a Hewlett-Packard D300 Digital Dispenser. Vemurafenib (PLX4032) was purchased from MedChem Express. PD98059, MK2206 and SB230580 were purchased from Selleck Chemicals. All compounds were dissolved in DMSO as 10-mM stock solutions for *in vitro* studies.

### Image quantification and registration

Plates were imaged with a × 10 objective using a Cytell Cell Imaging System (GE). All raw images are available on HMS-LINCS webpage (http://lincs.hms.harvard.edu/). Image segmentation and analysis were performed using ImageJ with the scripts provided in Supplementary Information. Hoechst images were converted to nuclear masks and region of interests (ROIs). The same ROIs were applied to images for all data channels (488/555/647) and the fluorescent intensities were obtained. The nuclear masks were then converted into RING ROIs outside the nuclei and used to quantify channels of interest. The intensity data generated by ImageJ were then passed to Matlab for further processing and analysis. Other image processing protocols would undoubtedly work equally well. For the details of morphometric analysis, see Supplementary Information.

For sequential imaging of CycIF, image registration was accomplished with ImageJ scripts (see Supplementary Information) and plugins (StackReg: http://bigwww.epfl.ch/thevenaz/stackreg/; MultiStackReg: http://bradbusse.net/downloads.html). Hoechst images from different cycles were inputted as reference images to generate registration information; the same registration information was used to transform images from other channels. The transformed images were compiled into multi-image stacks and the image segmentation and data retrieval were performed as described above. All ImageJ and Matlab scripts are available in the Supplementary Note 1 as well as on our website (http://lincs.hms.harvard.edu/lin-NatCommun-2015/)

### Data analysis using viSNE and Wanderlust

The viSNE and Wanderlust Matlab codes in the CYT package were obtained from the Pe’er lab webpage (http://www.c2b2.columbia.edu/danapeerlab/html/software.html). The raw data generated from CycIF was imported into FlowJo and converted to FCS files. FCS files were then used as input for both the viSNE and Wanderlust toolkits. For viSNE, each FCS file represented single-cell data from COLO858 cells treated with a single dose of vemurafenib. All data files were aggregated and used to generate viSNE diagrams. For Wanderlust, the same FCS files were normalized using the Wanderlust script with default parameters (L number=30; K number=5; number of landmarks=20; number of graphs=25; distance metric=cosine). Data from the DMSO-treated control sample were the starting point for the Wanderlust trajectory. Raw data for generating viSNE and Wanderlust plots are included in Supplementary Data 1.

## Additional information

**How to cite this article:** Lin, J.-R. *et al.* Highly multiplexed imaging of single cells using a high-throughput cyclic immunofluorescence method. *Nat. Commun.* 6:8390 doi: 10.1038/ncomms9390 (2015).

## Supplementary Material

Supplementary Information Supplementary Figures 1-9, Supplementary Tables 1-3 and Supplementary Note 1

Supplementary Data 1 The raw intensity data used in Figure 3. Each column re-presents a single-cell measurement from 8-channel CycIF, including p-H3, p-RB, p21, p-S6(S235/236), p-S6(S240/244), Ki-67 and two DAPI signals.
