# Supplementary material for: Highly multiplexed imaging of single cells using a high-throughput cyclic immunofluorescence method
Source: Nat Commun. 2015 Sep 24;6:8390. doi: 10.1038/ncomms9390 (PMC4587398; doi:10.1038/ncomms9390)
Supplement: Supplementary Information — Supplementary Figures 1-9, Supplementary Tables 1-3 and Supplementary Note 1 [file ncomms9390-s1.pdf]

Supplementary Figures

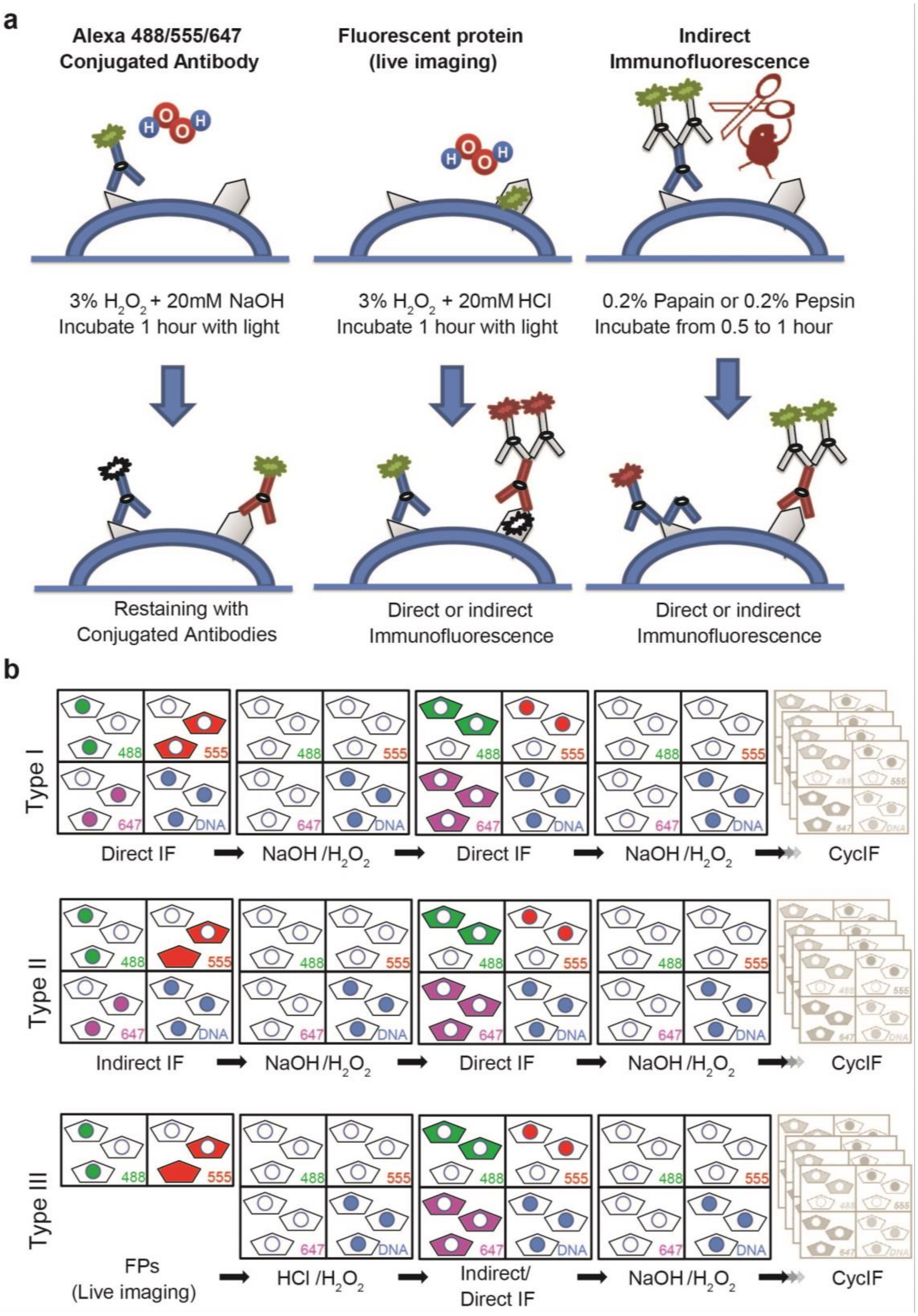

Supplementary Figure 1. CycIF methods and their variations. (a) Three different fluorophore inactivation methods used in CycIF. Chemical inactivation of fluorophores by using base-catalyzed oxidation for bleaching Alexa 488/555/647 fluorophores. For fluorescent proteins, an alternate acid-catalyzed peroxidation is used. Protease-mediated antibody stripping is used for indirect immunofluorescence to digest both primary/secondary antibodies. **(b)** Three types of CycIF workflows for increasing multiplicity. Typical CycIF (type I) uses only direct IF with fluorophore-conjugated antibodies. Type II CycIF combines indirect and direct IF, with indirect IF (primary/secondary antibodies) used once in the beginning. Type III CycIF uses a combination of indirect and direct IF at the end of live imaging of fluorescent proteins.

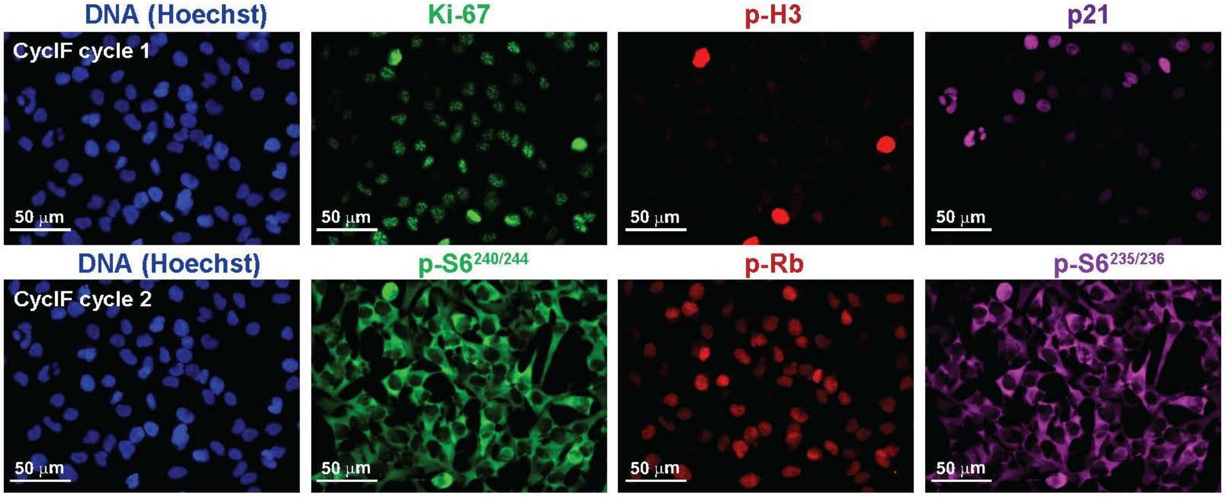

Supplementary Figure 2. Represented images of chemical inactivation CycIF. COLO858 melanoma cells were first stained with Alexa 488 conjugated Ki-67, Alexa 555 conjugated p-H3 and Alexa 647 conjugated p21 antibodies. Fluorophores were then inactivated and cells were re-stained with Alexa 488 conjugated p-S6$^{S240/244}$, Alexa 555 conjugated p-Rb and Alexa 647 conjugated p-S6$^{S235/236}$ antibodies. All images were acquired by GE Cytell imager as described in Methods.

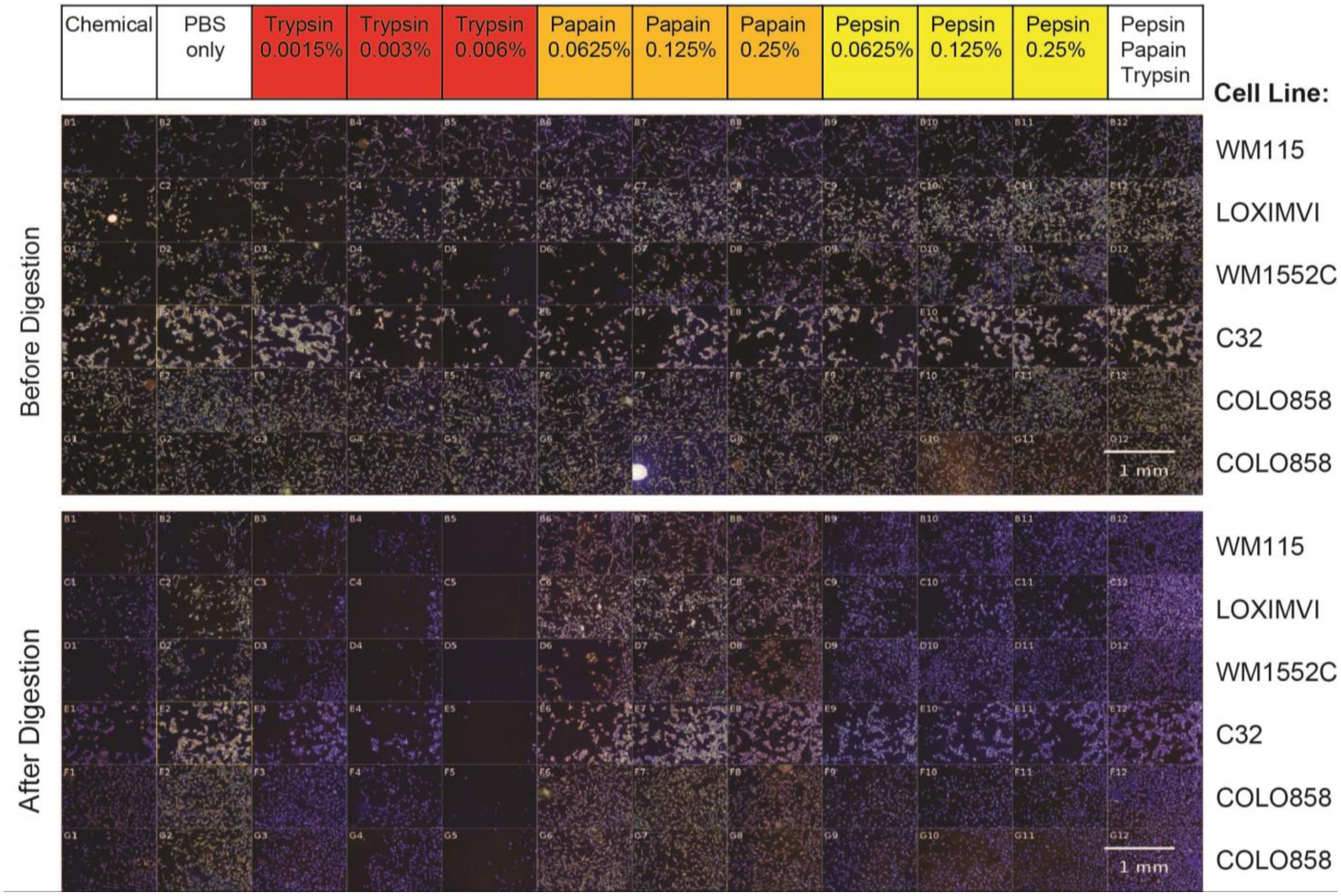

Supplementary Figure 3. **Optimization of the conditions for protease-mediated antibody stripping in five melanoma cell lines.** Different melanoma cell lines were seeded in different rows and stained with Hoechst (blue), $pS6^{S235/S236}$ (green), p-Rb (red) and p-$S6^{S240/S244}$ (magenta). Protease type, concentration and digestion time required for maximal fluorophore inactivation were balanced against cell loss for each individual cell line.

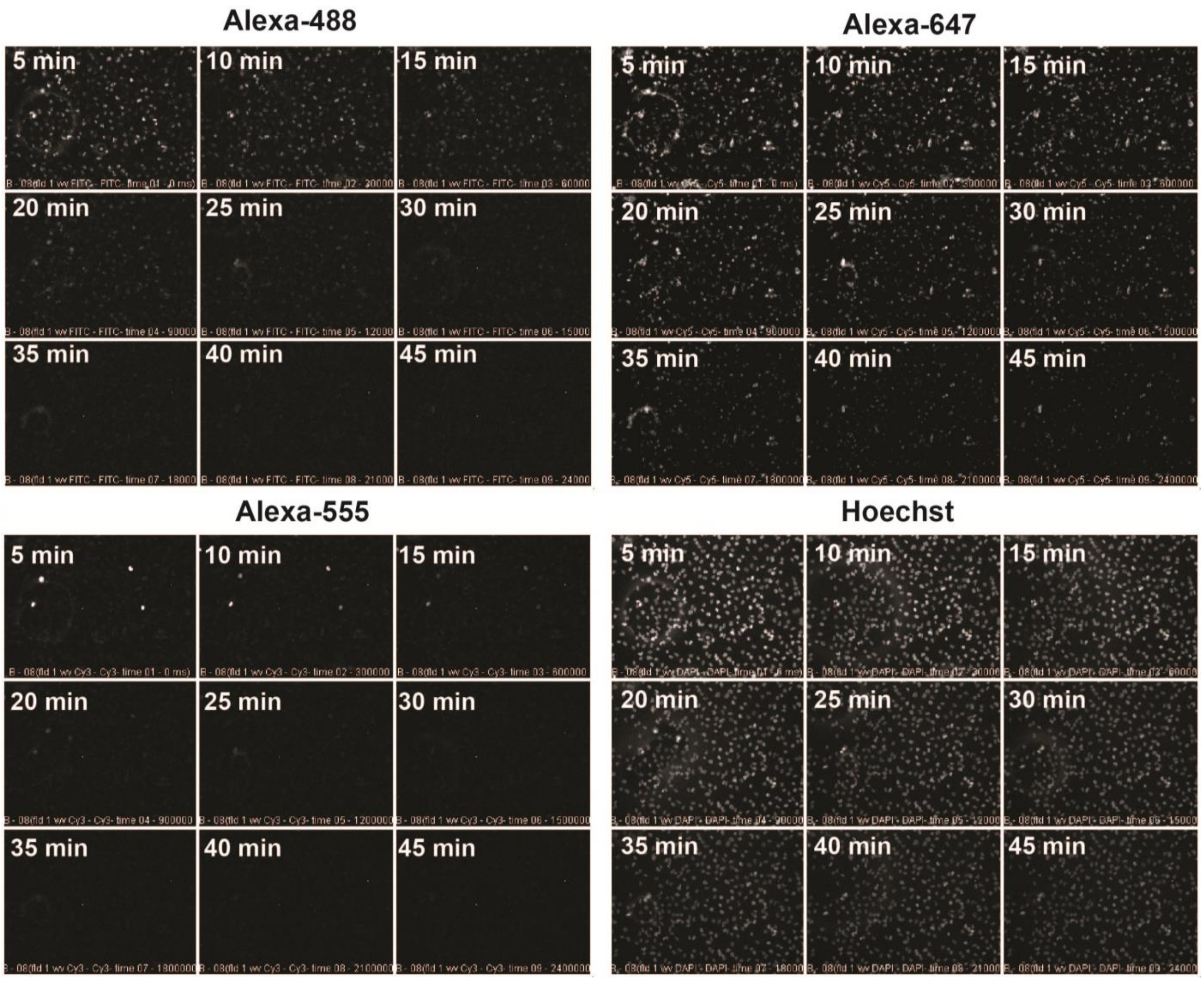

Supplementary Figure 4. Time-lapse imaging of the process of fluorophore inactivation. COLO858 cells were stained with Alexa 488-conjugated p-S6$^{S240/S244}$, Alexa 555-conjugated p-Rb and Alexa 647-conjugated p-S6$^{S235/S236}$ antibodies After staining, cells were incubated with 20 mM NaOH/3% H2O2 and imaged using a Cytell imager for 45 min (5 min per frame).

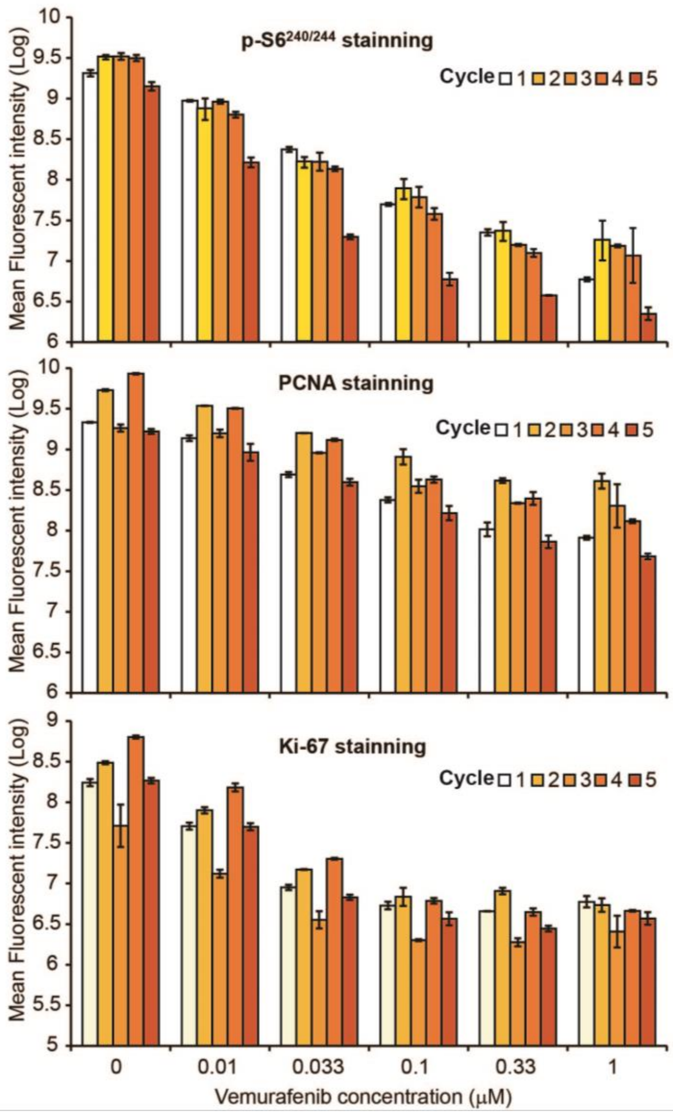

Supplementary Figure 5. Limited signal decay after fluorophore inactivation. Five-cycle CycIF was applied to COLO858 cells treated with increasing doses of vemurafenib in 96 wells in 5 different sets of replicates. Cells were stained with Alexa 488-conjugated p-S6$^{S240/244}$, PCNA and Ki-67 antibodies and cycled through different sets sequentially. The mean intensities of antibody staining from different cycles were quantified and plotted.

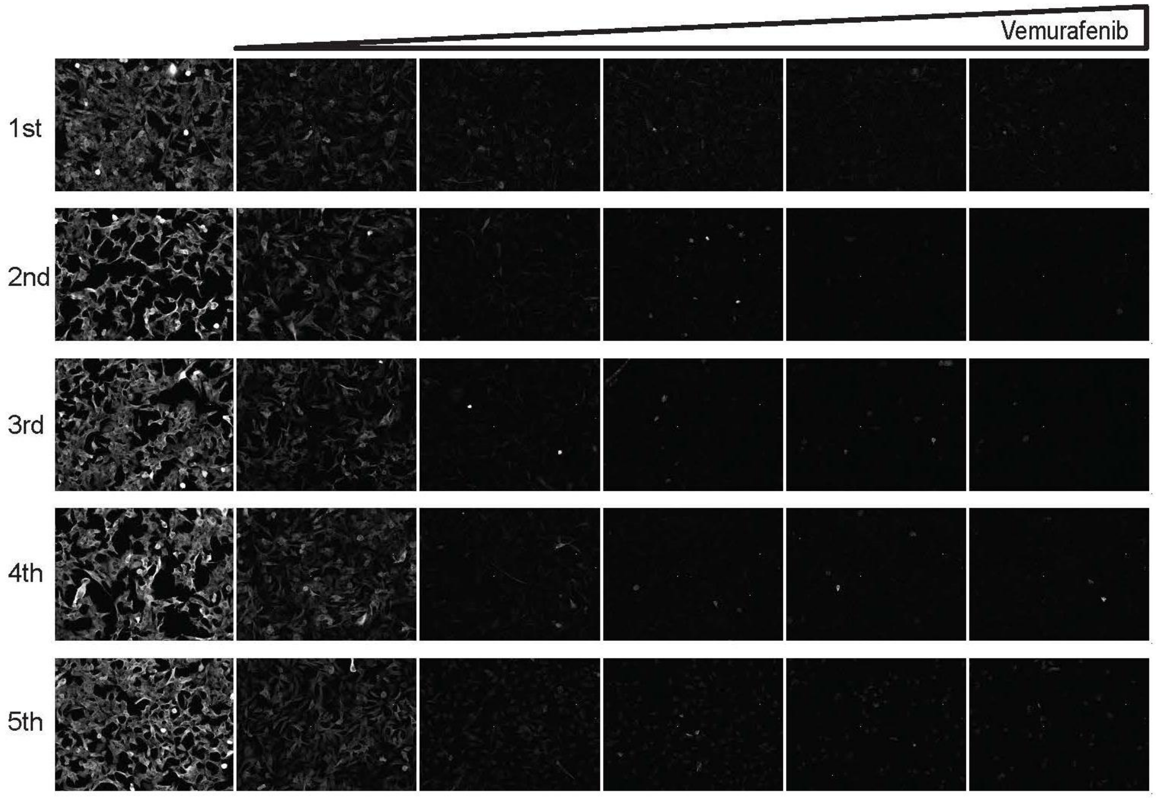

Supplementary Figure 6. p-ERK signals in five rounds of CycIF cycles. Five-cycle CycIF was applied to COLO858 cells treated with increasing doses of vemurafenib in 96 wells in 5 different sets of replicates. The Alexa-488 conjugated p-ERK antibodies were used for staining in 5 replicates and in different cycles. The representative images for different vemurafenib doses from different cycles are shown.

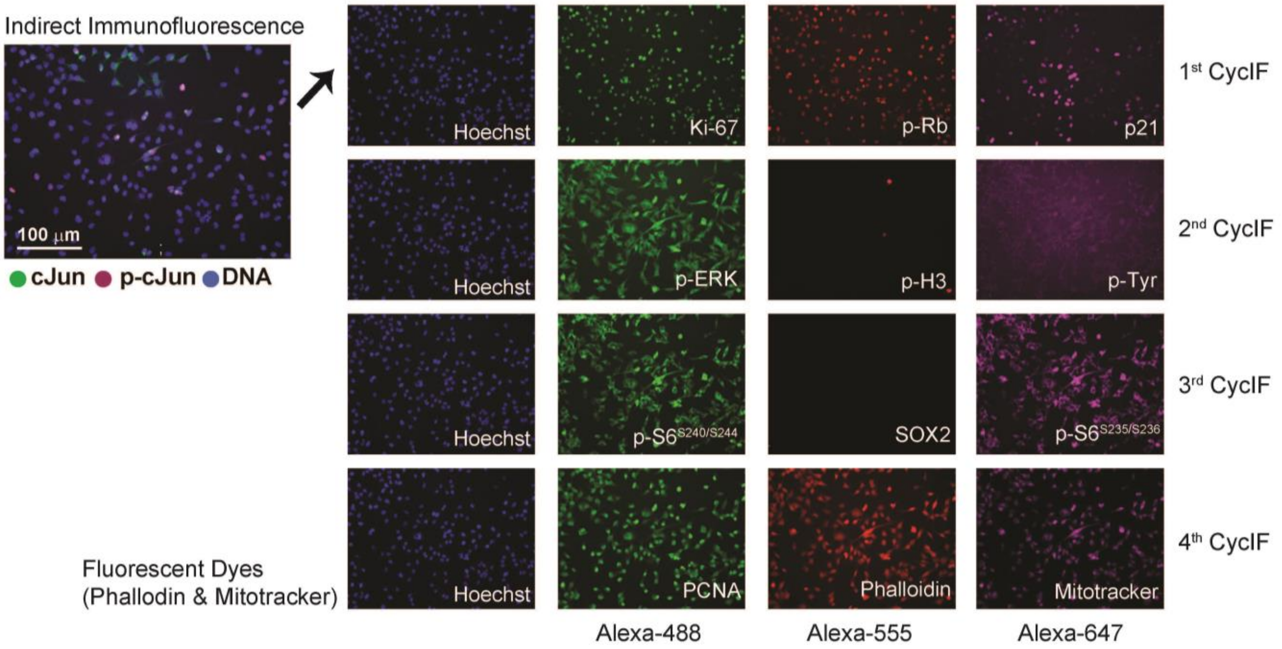

Supplementary Figure 7. Highly multiplexed single-cell imaging achieved by CycIF. CycIF combining indirect and direct IF was used sequentially to stain COLO858 cells. Cells were initially stained with c-Jun and p-cJun primary mouse and rabbit antibodies followed by Alexa 647-conjugated anti-mouse and Alexa 488-conjugated anti-rabbit secondary antibodies (left panel). Three cycles of CycIF with fluorophore-conjugated antibodies (cycle 1: Ki-67/p-Rb/p21; cycle 2: p-ERK/p-H3/p-Tyr; cycle 3: p-S6$^{S240/S244}$/SOX2/p-S6$^{S235/S236}$) were then applied. Finally, the fourth cycle was performed using fluorescent dyes (phalloidin & Mitotracker) for morphological analysis and Alexa 488-conjugated antibody for PCNA, a marker of cell proliferation.

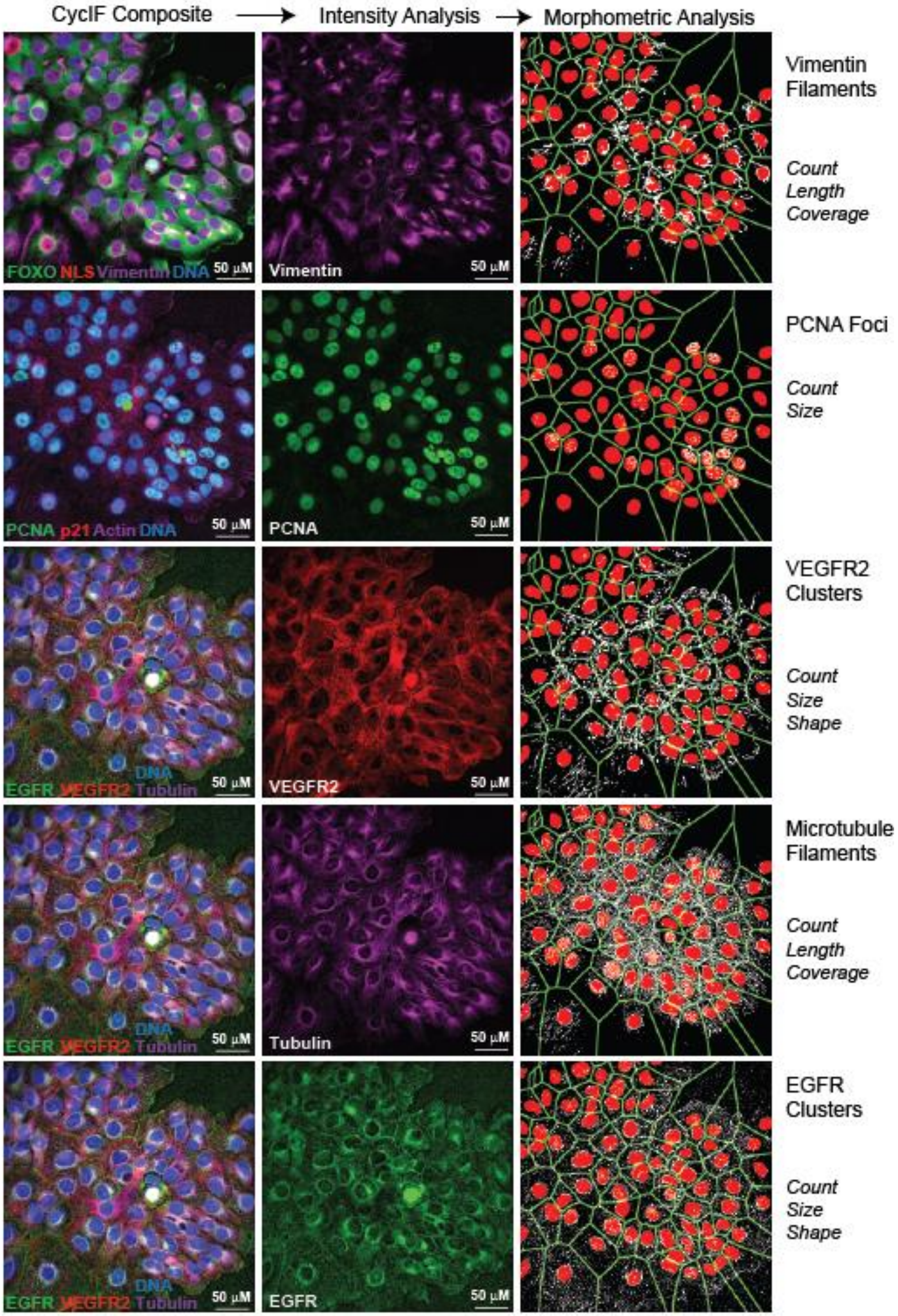
CycIF Composite
Intensity Analysis
Morphometric Analysis
FOXO NLS Vimentin DNA
50 μM
Vimentin
Vimentin Filaments
Count
Length
Coverage
PCNA p21 Actin DNA
PCNA
PCNA Foci
Count
Size
EGFR VEGFR2 DNA Tubulin
VEGFR2
VEGFR2 Clusters
Count
Size
Shape
Tubulin
Microtubule Filaments
Count
Length
Coverage
EGFR
EGFR Clusters
Count
Size
Shape

Supplementary Figure 8. Morphometric-feature extraction from CycIF images. Images from 3 rounds of CycIF staining were processed, segmented, and quantified in pixel intensity. Five IF signals, including PCNA, EGFR, VEGFR2, Vimentin and Tubulin were selected for feature extractions. Nuclear masks were generated from binarized Hoechst/DAPI staining. Filled and watershed functions were implemented to increase single-cell resolution. Cytoplasm/whole-cell masks were then generated through Voronoi transformation from nuclear masks.

For nuclear foci analysis, raw PCNA images were passed through an extreme local background subtraction to build binary puncta masks. Particle analysis was performed at the single-cell level and two morphometric features (foci count and foci size) were selected arbitrarily.

For cytoskeleton analysis, the raw Tubulin and Vimentin images were passed through an extreme local background subtraction and transformed into binary masks. The skeletonized function was then applied to the binary masks to generate final cytoskeleton masks. Particle analysis was performed at the single-cell level and three morphometric features (filament count, filament length and filament coverage) were selected arbitrarily.

For receptor/vesicle analysis, raw EGFR and VEGFR2 images were passed through an extreme local background subtraction and transformed into binary masks. Outliers or noise from image processing were then removed by de-speckle transformation. Particle analysis was performed at the single-cell level and four morphometric features (cluster count, cluster length, cluster Feret diameter and cluster Feret angle) were selected arbitrarily. For details, see the provided ImageJ script.

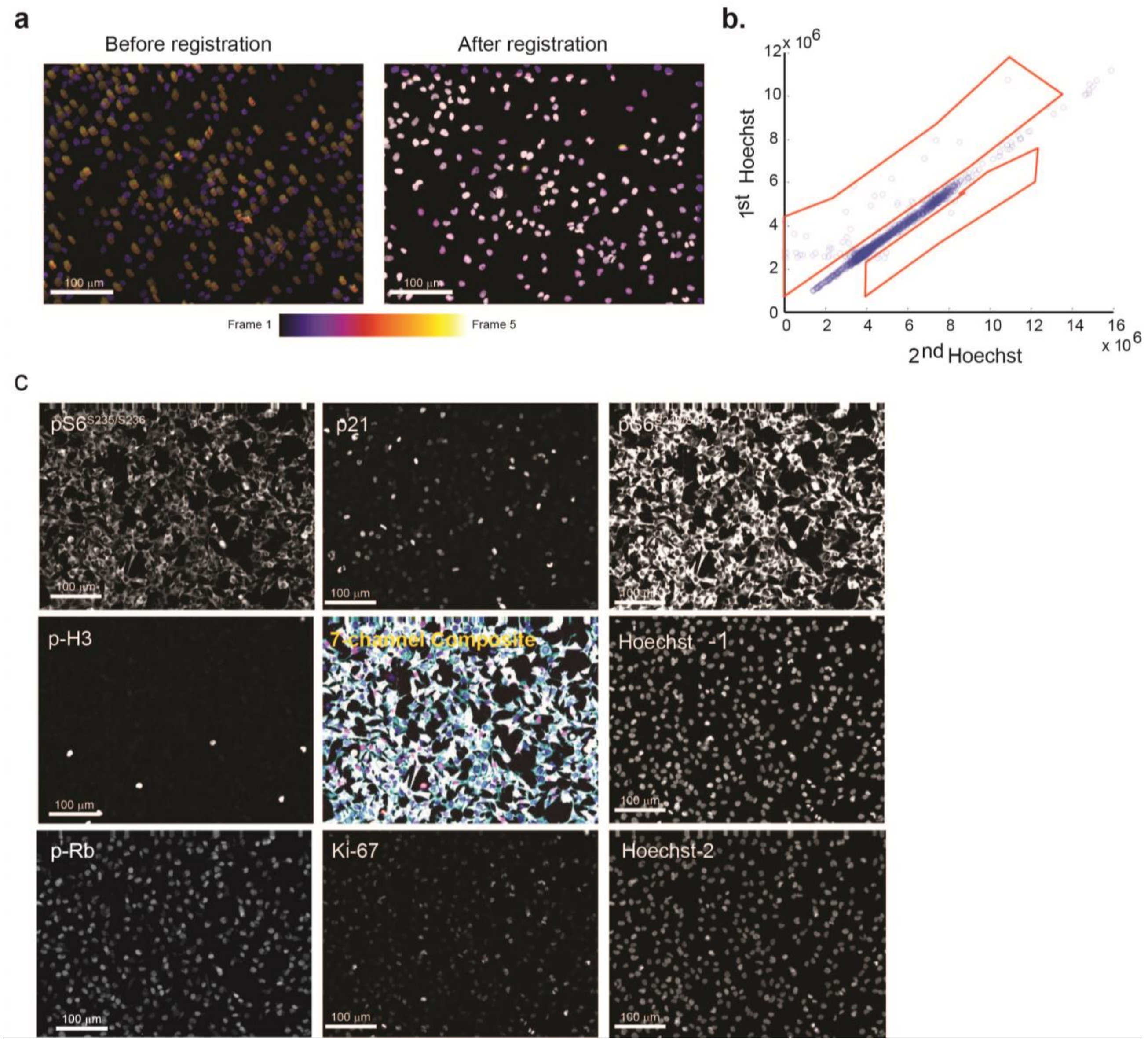

Supplementary Figure 9. Image registration and analysis following CycIF. (a,b) Image registration and analysis by ImageJ. The Hoechst images from five different cycles of CycIF (before and after registration) are overlaid and colored. (a). Hoechst staining was used to gate cell gain or loss between CycIF cycles. Two subsequent CycIF Hoechst images from the same samples were analyzed and single-cell Hoechst intensities are plotted. (b). The diagonal cells are the matched population and the red boxes indicate either gained or lost cells between the two CycIF cycles. **(c)** Source images and 7-channels composite for the experiments described in Fig. 3.

Supplementary Table 1: The list of fluorophore-conjugated antibodies used in this study. All antibodies were obtained from Cell Signaling Technologies.

Alexa-488 Conjugated	Alexa-555 Conjugated	Alexa-647 Conjugated
p-ERK1/2$^{T202/Y204}$<br>(rabbit mAb; 1:200; #4344)	p-Rb$^{S807/S811}$<br>(rabbit mAb; 1:400; #8957)	p21Waf1/Cip1<br>(rabbit mAb; 1:200; #8587)
EGFR<br>(rabbit mAb; 1:400; #5616)	p-Histone H3^{S10}<br>(rabbit mAb; 1:400; #3475)	pS6$^{S235/S236}$<br>(rabbit mAb; 1:200; #4851)
EpCAM<br>(mouse mAb; 1:100; #5198)	pan-keratin<br>(mouse mAb; 1:100; #3478)	beta-tubulin<br>(rabbit mAb; 1:100; #3624)
Lamin A/C<br>(mouse mAb; 1:200; #8617)	p-Histone H2A.X^{S139}<br>(rabbit mAb; 1:100; #8228)	p-Tyrosine<br>(mouse mAb; 1:100; #9415).
p-S6$^{S240/S244}$<br>(rabbit mAb; 1:200; #5018)	Sox2<br>(rabbit mAb; 1:200; #5179)	
PCNA<br>(mouse mAb; 1:400; #8580)	β-actin<br>(rabbit mAb; 1:200; #8046)	

Supplementary Table 2: Abbreviations

IF	Immunofluorescence
IR	Infrared
FP	Fluorescent Protein
ERK	Extracellular signal-Regulated Kinase
Rb	Retinoblastoma protein
EGFR	Epidermal Growth Factor Receptor
PCNA	Proliferating Cell Nuclear Antigen
S6	Ribosomal protein S6
RSK	Ribosomal S6 Kinase
mTOR	mammalian Target Of Rapamycin
VEGFR2	Vascular Endothelial Growth Factor Receptor 2

Supplementary Table 3: Tested fluorophore-conjugated antibodies for CycIF

Alexa-488/FITC Conjugated	Alexa-555/TRITC Conjugated	Alexa-647 Conjugated
p-ERK1/ (CST #4344)	p-Rb S807/S811 (CST #8957)	p21Waf1/Cip1 (CST #8587)
EGFR (CST #5616)	p-Histone H3 S10 (CST #3475)	pS6 S235/S236 (CST #4851)
EpCAM (CST #5198)	pan-keratin (CST #3478)	beta-tubulin (CST #3624)
Lamin A/C (CST #8617)	p-Histone H2A.XS139 (CST #8228)	p-Tyrosine (CST#9415)
p-S6 S240/S244 (CST #5018)	Sox2 (CST #5179)	beta-Catenin (CST #4627)
PCNA (CST #8580)	β-actin (CST #8046)	mTOR (CST #5048)
p-cJUN (CST #12714)	Oct-4A (CST #4439)	pan-Akt (CST #5186)
Ki-67 (CST #11822)	VEGFR2 (CST #12872)	FOXO3a (AB196539) N/D
Cyclin D1 (AB190194)	LC3A/B (CST #13173)	p65NFkB (AB190589)
p-CREB (CST #9187)	ActinRed (Invitrogen R371112)	p27 (AB194234)
p-HSP27 (CST #12172)	cMyc (SC-40)	p75NTR (AB195180)
cJUN (AB193780)	cPARP (CST #6894)	p-H2ax S139 (CST #9270)
E-Cadherin (CST #3199)	Vimentin (CST #9855)	Vimentin (CST #9856)
B220 (CD45R, Biolegend)	pS6 S235/S236 (CST #3985)	Her2 (Biolegend 324412)
Cyclin B1 (SC-752)	p21Waf1/Cip1 (CST #8493)	CD45 (Biolegend 304020)
Bax (Biolegend 633603)	p-AuroraABC (CST #13464)	Bcl-2 (Biolegend 658705)
cdc2/CDK1 (sc-54)	S6-total (CST #6989)	p-H2ax S139 (Biolegend 613407)

Color code and abbreviations

Validated in several cell lines	Validated in at least one cell lines	Non-validated
CST : Cell Signaling Technology	AB: Abcam	SC: Santa Cruz

Supplementary note 1

Image segmentation and quantification (ImageJ)

```
//Simple script for measure Cytell results (four channels);
//Jerry  2014/10/09

path = getDirectory("Select the image directory");
start = getTime;

// generate nuclear mask

setBatchMode(true);

run("Image Sequence...", "open=["+path+"B - 2(fld 1 wv DAPI - DAPI).TIF] file=DAPI sort");

run("Subtract Background...", "rolling=50 stack");
run("Enhance Contrast...", "saturated=0.4");
rename("DAPI");
run("Duplicate...", "title=mask duplicate range=1-540");
setOption("BlackBackground", false);
run("Make Binary", "method=Li background=Default calculate");
run("Fill Holes", "stack");
run("Watershed", "stack");

run("Set Scale...", "distance=0 known=0 pixel=1 unit=pixel global");
run("Set Measurements...", "area mean standard min centroid perimeter shape integrated median
skewness kurtosis area_fraction stack display add redirect=None decimal=3");
run("Analyze Particles...", "size=100-1000 circularity=0.10-1.00 clear include add stack");
close();

print("Buidling Mask",(getTime-start)/1000);

//measure DAPI channel

roiManager("Show None");
selectWindow("DAPI");
roiManager("Measure");
saveAs("Results", path+"results-DAPI.csv");
run("Clear Results");
selectWindow("DAPI");
close();

print("Measuring DAPI",(getTime-start)/1000);

// Measure Cy3 channel

run("Image Sequence...", "open=["+path+"B - 2(fld 1 wv Cy3 - Cy3).TIF] disable_these file=Cy3
sort");

rename("Cy3");
run("Subtract Background...", "rolling=50 stack");
run("Enhance Contrast...", "saturated=0.4");
roiManager("Measure");

saveAs("Results", path+"Results-Cy3.csv");
run("Clear Results");
selectWindow("Cy3");
close();
```

```
print("Measuring Cy3",(getTime-start)/1000);

// Measure Cy5 channel

run("Image Sequence...", "open=["+path+"B - 2(fld 1 wv Cy5 - Cy5).TIF] file=Cy5 sort");

rename("Cy5");
run("Subtract Background...", "rolling=50 stack");
run("Enhance Contrast...", "saturated=0.4");
roiManager("Measure");
saveAs("Results",path+"Results-Cy5.csv");
run("Clear Results");
selectWindow("Cy5");
close();

print("Measuring Cy5",(getTime-start)/1000);

// Measure FITC channel

run("Image Sequence...", "open=["+path+"B - 2(fld 1 wv Cy5 - Cy5).TIF] file=FITC sort");

rename("FITC")
run("Subtract Background...", "rolling=50 stack");
run("Enhance Contrast...", "saturated=0.4");
roiManager("Measure");
saveAs("Results", path+"Results-FITC.csv");
run("Clear Results");
selectWindow("FITC");
close();

print("Measuring FITC",(getTime-start)/1000);

setBatchMode(false);
```

Image registration and measurement (ImageJ)

```
//script for merge CycIF imaging & measure single-cell data
//Jerry 2014/12/28
// for Cytell imageing

row = newArray(" ","A","B","C","D","E","F","G","H");
col =newArray("00","01","02","03","04","05","06","07","08","09","10","11","12");

start = getTime;

while (nImages>0) {
          selectImage(nImages);
          close();
}

if (isOpen("ROI Manager")) {
      selectWindow("ROI Manager");
      run("Close");
}

setBatchMode(true);

for (r=2;r<8;r++)          //row array
```

```
{
for (c=2;c<12;c++)         //column array
{
for (f=1;f<10;f++)         //filed
{

run("Image Sequence...", "open=[C:\\CycIF\\1st\\B - 02(fld 01 wv Cy3 - Cy3).tif]
file=["+row[r]+" - "+col[c]+"(fld 0"+f+"] sort");
run("Stack to Images");

run("Image Sequence...", "open=[C:\\CycIF\\2nd\\B - 02(fld 01 wv Cy3 - Cy3).tif]
file=["+row[r]+" - "+col[c]+"(fld 0"+f+"] sort");
run("Stack to Images");

run("Image Sequence...", "open=[C:\\CycIF\\3rd\\B - 02(fld 01 wv Cy3 - Cy3).tif]
file=["+row[r]+" - "+col[c]+"(fld 0"+f+"] sort");
run("Stack to Images");

run("Image Sequence...", "open=[C:\\CycIF\\4th\\B - 02(fld 01 wv Cy3 - Cy3).tif]
file=["+row[r]+" - "+col[c]+"(fld 0"+f+"] sort");
run("Stack to Images");

run("Image Sequence...", "open=[C:\\CycIF\\5th\\B - 02(fld 01 wv Cy3 - Cy3).tif]
file=["+row[r]+" - "+col[c]+"(fld 0"+f+"] sort");
run("Stack to Images");

run("Images to Stack", "name=DAPI title=DAPI");
run("Subtract Background...", "rolling=50 stack");

run("Images to Stack", "name=FITC title=FITC");
run("Subtract Background...", "rolling=50 stack");

run("Images to Stack", "name=Cy5 title=Cy5");
run("Subtract Background...", "rolling=50 stack");

run("Images to Stack", "name=Cy3 title=Cy3");
run("Subtract Background...", "rolling=50 stack");


run("MultiStackReg", "stack_1=DAPI action_1=Align file_1=c:\\CycIF\\reg1.txt stack_2=None
action_2=Ignore file_2=[] transformation=[Rigid Body] save");

run("MultiStackReg", "stack_1=FITC action_1=[Load Transformation File]
file_1=c:\\CycIF\\reg1.txt stack_2=None action_2=Ignore file_2=[] transformation=[Rigid
Body]");

run("MultiStackReg", "stack_1=Cy5 action_1=[Load Transformation File]
file_1=c:\\CycIF\\reg1.txt stack_2=None action_2=Ignore file_2=[] transformation=[Rigid
Body]");

run("MultiStackReg", "stack_1=Cy3 action_1=[Load Transformation File]
file_1=c:\\CycIF\\reg1.txt stack_2=None action_2=Ignore file_2=[] transformation=[Rigid
Body]");

selectWindow("DAPI");
run("Stack to Images");
selectWindow("DAPI-0001");
run("Duplicate...", "title=MASK");
setOption("BlackBackground", false);
run("Make Binary");
run("Fill Holes");
run("Dilate");
run("Watershed");
```

```
run("Analyze Particles...", "size=100-1500 pixel circularity=0.10-1.00 exclude clear include
add");
selectWindow("MASK");
close();
selectWindow("Cy3");
run("Stack to Images");
selectWindow("Cy5");
run("Stack to Images");
selectWindow("FITC");
run("Stack to Images");

run("Images to Stack", "name="+row[r]+col[c]+"_fld"+f+" title=[] use");
roiManager("Measure");

for (i=1;i<20;i++)         //measure each frames (expect the first frame)
{
run("Next Slice [>]");
roiManager("Measure");
}

saveAs("Results", "C:\\CycIF\\Results-"+row[r]+col[c]+"fld0"+f+".txt");
run("Clear Results");
close();
print("Well ",row[r],"-",col[c],"-",f," finisehd:",(getTime-start)/1000);
} //for f
} //for c
} //for r

setBatchMode(false);
```

Morphometric analysis (ImageJ)

```
// Obtain imaging features (beta)
// Jerry 2015/05/31
// for 5-ch images (DAPI, Vimentin, Tubulin, EGFR, VEGFR2, PCNA)
// generate nucleus and cytoplasmic masks
selectWindow("DAPI");
run("Duplicate...", " ");
setOption("BlackBackground", false);
run("Make Binary");
run("Fill Holes");
run("Watershed");
rename("Nucleus");
run("Duplicate...", "title=new");
run("Voronoi");
run("Multiply...", "value=50");
run("Multiply...", "value=2");
run("Make Binary");
close();
selectWindow("Nucleus");
run("Duplicate...", "title=new");
run("Voronoi");
run("Multiply...", "value=255");
setOption("BlackBackground", false);
run("Make Binary");
```

```
run("Dilate");
rename("Cyto");
run("Duplicate...", "title=new");
run("Invert");
rename("mask");
run("Analyze Particles...", "size=2000-50000 pixel display clear summarize add");
selectWindow("mask");
run("Close");

run("Merge Channels...", "c1=Nucleus c2=Cyto create keep");
rename("Cyto-Nuc");
run("Labels...", "color=yellow font=14 show bold");
run("From ROI Manager");

//Generate PCNA mask
selectWindow("PCNA");
run("Subtract Background...", "rolling=50");
run("Duplicate...", "title=PCNA-F");
run("Subtract Background...", "rolling=4");
run("Subtract...", "value=200");
run("Multiply...", "value=10");
setOption("BlackBackground", false);
run("Make Binary");
run("Dilate");
run("Merge Channels...", "c1=Nucleus c2=Cyto c5=PCNA-F create keep");
rename("Composite-PCNA");
run("From ROI Manager");

//Generate Tubulin Mask
selectWindow("Tubulin");
run("Subtract Background...", "rolling=50");
run("Duplicate...", "title=Tubulin-F");
run("Subtract Background...", "rolling=4 disable");
run("Make Binary");
run("Skeletonize");
run("Dilate");
run("Merge Channels...", "c1=Nucleus c2=Cyto c5=Tubulin-F create keep");
rename("Composite-Tubulin");
run("From ROI Manager");

//Generate Vimentin Mask
selectWindow("Vimentin");
run("Subtract Background...", "rolling=50");
run("Duplicate...", "title=Vimentin-F");
run("Subtract Background...", "rolling=4 disable");
setOption("BlackBackground", false);
run("Make Binary");
run("Despeckle");
run("Skeletonize");
run("Dilate");
run("Merge Channels...", "c1=Nucleus c2=Cyto c5=Vimentin-F create keep");
rename("Composite-Vimentin");
run("From ROI Manager");

//Generate VEGFR2 mask
selectWindow("VEGFR2");
run("Subtract Background...", "rolling=50");
run("Duplicate...", "title=VEGFR2-F");
run("Subtract Background...", "rolling=4 disable");
run("Subtract...", "value=100");
setOption("BlackBackground", false);
run("Make Binary");
run("Despeckle");
```

```
run("Merge Channels...", "c1=Nucleus c2=Cyto c5=VEGFR2-F create keep");
rename("Composite-VEGFR2");
run("From ROI Manager");

//Generate EGFR mask
selectWindow("EGFR");
run("Subtract Background...", "rolling=50");
run("Duplicate...", "title=EGFR-F");
run("Subtract Background...", "rolling=4 disable");
run("Subtract...", "value=100");
run("Make Binary");
run("Despeckle");
run("Dilate");
run("Merge Channels...", "c1=Nucleus c2=Cyto c5=EGFR-F create keep");
rename("Composite-EGFR");
run("From ROI Manager");


//Measure PCNA
selectWindow("PCNA-F");
run("Set Measurements...", "area mean standard min centroid perimeter shape feret's
integrated median skewness kurtosis display add redirect=None decimal=3");
nroi = roiManager("count");
for (i=0; i<nroi; i++) {
    roiManager("select",i);
    run("Analyze Particles...", "size=1-Infinity circularity=0.00-1.00 show=Nothing
summarize");
 }
roiManager("Deselect");
run("Set Measurements...", "area mean standard min centroid perimeter shape feret's
integrated median skewness kurtosis display add redirect=None decimal=3");
selectWindow("PCNA");
roiManager("Measure");
selectWindow("Results");
saveAs("Results", "C:\\Users\\JLin\\Desktop\\Results-PCNA.txt");
run("Close");
selectWindow("Summary");
saveAs("Results", "C:\\Users\\JLin\\Desktop\\Summary-PCNA.txt");
run("Close");

//Measure EGFR
selectWindow("EGFR-F");
run("Set Measurements...", "area mean standard min centroid perimeter shape feret's
integrated median skewness kurtosis display add redirect=None decimal=3");
nroi = roiManager("count");
for (i=0; i<nroi; i++) {
    roiManager("select",i);
    run("Analyze Particles...", "size=1-Infinity circularity=0.00-1.00 show=Nothing
summarize");
 }
roiManager("Deselect");
run("Set Measurements...", "area mean standard min centroid perimeter shape feret's
integrated median skewness kurtosis display add redirect=None decimal=3");
selectWindow("EGFR");
roiManager("Measure");
selectWindow("Results");
saveAs("Results", "C:\\Users\\JLin\\Desktop\\Results-EGFR.txt");
run("Close");
selectWindow("Summary");
saveAs("Results", "C:\\Users\\JLin\\Desktop\\Summary-EGFR.txt");
run("Close");

//Measure VEGFR2
```

```
selectWindow("VEGFR2-F");
run("Set Measurements...", "area mean standard min centroid perimeter shape feret's
integrated median skewness kurtosis display add redirect=None decimal=3");
nroi = roiManager("count");
for (i=0; i<nroi; i++) {
    roiManager("select",i);
    run("Analyze Particles...", "size=1-Infinity circularity=0.00-1.00 show=Nothing
summarize");
 }
roiManager("Deselect");
run("Set Measurements...", "area mean standard min centroid perimeter shape feret's
integrated median skewness kurtosis display add redirect=None decimal=3");
selectWindow("VEGFR2");
roiManager("Measure");
selectWindow("Results");
saveAs("Results", "C:\\Users\\JLin\\Desktop\\Results-VEGFR2.txt");
run("Close");
selectWindow("Summary");
saveAs("Results", "C:\\Users\\JLin\\Desktop\\Summary-VEGFR2.txt");
run("Close");

//Measure Tubulin
selectWindow("Tubulin-F");
run("Set Measurements...", "area mean standard min centroid perimeter shape feret's
integrated median skewness kurtosis display add redirect=None decimal=3");
nroi = roiManager("count");
for (i=0; i<nroi; i++) {
    roiManager("select",i);
    run("Analyze Particles...", "size=1-Infinity circularity=0.00-1.00 show=Nothing
summarize");
 }
roiManager("Deselect");
run("Set Measurements...", "area mean standard min centroid perimeter shape feret's
integrated median skewness kurtosis display add redirect=None decimal=3");
selectWindow("Tubulin");
roiManager("Measure");
selectWindow("Results");
saveAs("Results", "C:\\Users\\JLin\\Desktop\\Results-Tubulin.txt");
run("Close");
selectWindow("Summary");
saveAs("Results", "C:\\Users\\JLin\\Desktop\\Summary-Tubulin.txt");
run("Close");

//Measure Vimentin
selectWindow("Vimentin-F");
run("Set Measurements...", "area mean standard min centroid perimeter shape feret's
integrated median skewness kurtosis display add redirect=None decimal=3");
nroi = roiManager("count");
for (i=0; i<nroi; i++) {
    roiManager("select",i);
    run("Analyze Particles...", "size=1-Infinity circularity=0.00-1.00 show=Nothing
summarize");
 }
roiManager("Deselect");
run("Set Measurements...", "area mean standard min centroid perimeter shape feret's
integrated median skewness kurtosis display add redirect=None decimal=3");
selectWindow("Vimentin");
roiManager("Measure");
selectWindow("Results");
saveAs("Results", "C:\\Users\\JLin\\Desktop\\Results-Vimentin.txt");
run("Close");
selectWindow("Summary");
saveAs("Results", "C:\\Users\\JLin\\Desktop\\Summary-Vimentin.txt");
```

```
run("Close");
```

Data importing (Matlab)

```
%% Import data from text file.
% Script for importing data from the following text file:
%
%    Z:\sorger\data\Cytell\Jerry\Jerry-96well(3603)-4channels-9sites_20140922-pAKT2-
20140917\results-DAPI.csv
%
% To extend the code to different selected data or a different text file,
% generate a function instead of a script.

% Auto-generated by MATLAB on 2014/10/10 12:18:18

%% Initialize variables.
mypath = uigetdir('Z:\sorger\data\Cytell\Jerry');


%% Initialize variables.
filename = strcat(mypath,'\results-DAPI.csv');

delimiter = ',';
startRow = 2;

%% Format string for each line of text:
%   column1: double (%f)
%      column2: text (%s)
%   column3: double (%f)
%      column4: double (%f)
%   column5: double (%f)
%      column6: double (%f)
%   column7: double (%f)
%      column8: double (%f)
%   column9: double (%f)
%      column10: double (%f)
%   column11: double (%f)
%      column12: double (%f)
%   column13: double (%f)
%      column14: double (%f)
%   column15: double (%f)
%      column16: double (%f)
%   column17: double (%f)
%      column18: double (%f)
%   column19: double (%f)
%      column20: double (%f)
%   column21: double (%f)
% For more information, see the TEXTSCAN documentation.
formatSpec = '%f%s%f%f%f%f%f%f%f%f%f%f%f%f%f%f%f%f%f%f%f%[^\n\r]';

%% Open the text file.
fileID = fopen(filename,'r');

%% Read columns of data according to format string.
% This call is based on the structure of the file used to generate this
% code. If an error occurs for a different file, try regenerating the code
% from the Import Tool.
dataArray = textscan(fileID, formatSpec, 'Delimiter', delimiter, 'EmptyValue'
,NaN,'HeaderLines' ,startRow-1, 'ReturnOnError', false);
```

```
%% Close the text file.
fclose(fileID);

%% Post processing for unimportable data.
% No unimportable data rules were applied during the import, so no post
% processing code is included. To generate code which works for
% unimportable data, select unimportable cells in a file and regenerate the
% script.

%% Create output variable
resultsDAPI = table(dataArray{1:end-1}, 'VariableNames',
{'VarName1','Well','Area','Mean','StdDev','Min','Max','X','Y','Perim','Circ','IntDen','Median
','Skew','Kurt','VarName16','RawIntDen','Slice','AR','Round','Solidity'});
resultsDAPI.Well = cellfun(@(x) x(length(x)-28:length(x)-
23),resultsDAPI.Well,'UniformOutput',false);

%% Clear temporary variables

clearvars filename delimiter startRow formatSpec fileID dataArray ans;

%% Import data from text file.
% Script for importing data from the following text file:
%
%    Z:\sorger\data\Cytell\Jerry\Jerry-96well(3603)-4channels-9sites_20140922-pAKT2-
20140917\results-FITC.csv
%
% To extend the code to different selected data or a different text file,
% generate a function instead of a script.

% Auto-generated by MATLAB on 2014/10/10 12:18:18

%% Initialize variables.
filename = strcat(mypath,'\results-FITC.csv');
delimiter = ',';
startRow = 2;

%% Format string for each line of text:
%   column1: double (%f)
%      column2: text (%s)
%   column3: double (%f)
%      column4: double (%f)
%   column5: double (%f)
%      column6: double (%f)
%   column7: double (%f)
%      column8: double (%f)
%   column9: double (%f)
%      column10: double (%f)
%   column11: double (%f)
%      column12: double (%f)
%   column13: double (%f)
%      column14: double (%f)
%   column15: double (%f)
%      column16: double (%f)
%   column17: double (%f)
%      column18: double (%f)
%   column19: double (%f)
%      column20: double (%f)
%   column21: double (%f)
% For more information, see the TEXTSCAN documentation.
formatSpec = '%f%s%f%f%f%f%f%f%f%f%f%f%f%f%f%f%f%f%f%f%f%[^\n\r]';

%% Open the text file.
```

```
fileID = fopen(filename,'r');

%% Read columns of data according to format string.
% This call is based on the structure of the file used to generate this
% code. If an error occurs for a different file, try regenerating the code
% from the Import Tool.
dataArray = textscan(fileID, formatSpec, 'Delimiter', delimiter, 'EmptyValue'
,NaN,'HeaderLines' ,startRow-1, 'ReturnOnError', false);

%% Close the text file.
fclose(fileID);

%% Post processing for unimportable data.
% No unimportable data rules were applied during the import, so no post
% processing code is included. To generate code which works for
% unimportable data, select unimportable cells in a file and regenerate the
% script.

%% Create output variable
resultsFITC = table(dataArray{1:end-1}, 'VariableNames',
{'VarName1','Well','Area','Mean','StdDev','Min','Max','X','Y','Perim','Circ','IntDen','Median
','Skew','Kurt','VarName16','RawIntDen','Slice','AR','Round','Solidity'});
resultsFITC.Well = cellfun(@(x) x(length(x)-28:length(x)-
23),resultsFITC.Well,'UniformOutput',false);
%% Clear temporary variables

clearvars filename delimiter startRow formatSpec fileID dataArray ans;

%% Import data from text file.
% Script for importing data from the following text file:
%
%    Z:\sorger\data\Cytell\Jerry\Jerry-96well(3603)-4channels-9sites_20140922-pAKT2-
20140917\results-Cy3.csv
%
% To extend the code to different selected data or a different text file,
% generate a function instead of a script.

% Auto-generated by MATLAB on 2014/10/10 12:18:18

%% Initialize variables.
filename = strcat(mypath,'\results-Cy3.csv');
delimiter = ',';
startRow = 2;

%% Format string for each line of text:
%   column1: double (%f)
%      column2: text (%s)
%   column3: double (%f)
%      column4: double (%f)
%   column5: double (%f)
%      column6: double (%f)
%   column7: double (%f)
%      column8: double (%f)
%   column9: double (%f)
%      column10: double (%f)
%   column11: double (%f)
%      column12: double (%f)
%   column13: double (%f)
%      column14: double (%f)
%   column15: double (%f)
%      column16: double (%f)
%   column17: double (%f)
%      column18: double (%f)
```

```
%   column19: double (%f)
%      column20: double (%f)
%   column21: double (%f)
% For more information, see the TEXTSCAN documentation.
formatSpec = '%f%s%f%f%f%f%f%f%f%f%f%f%f%f%f%f%f%f%f%f%f%[^\n\r]';

%% Open the text file.
fileID = fopen(filename,'r');

%% Read columns of data according to format string.
% This call is based on the structure of the file used to generate this
% code. If an error occurs for a different file, try regenerating the code
% from the Import Tool.
dataArray = textscan(fileID, formatSpec, 'Delimiter', delimiter, 'EmptyValue'
,NaN,'HeaderLines' ,startRow-1, 'ReturnOnError', false);

%% Close the text file.
fclose(fileID);

%% Post processing for unimportable data.
% No unimportable data rules were applied during the import, so no post
% processing code is included. To generate code which works for
% unimportable data, select unimportable cells in a file and regenerate the
% script.

%% Create output variable
resultsCy3 = table(dataArray{1:end-1}, 'VariableNames',
{'VarName1','Well','Area','Mean','StdDev','Min','Max','X','Y','Perim','Circ','IntDen','Median
','Skew','Kurt','VarName16','RawIntDen','Slice','AR','Round','Solidity'});
resultsCy3.Well = cellfun(@(x) x(length(x)-26:length(x)-
21),resultsCy3.Well,'UniformOutput',false);
%% Clear temporary variables


clearvars filename delimiter startRow formatSpec fileID dataArray ans;

%% Import data from text file.
% Script for importing data from the following text file:
%
%    Z:\sorger\data\Cytell\Jerry\Jerry-96well(3603)-4channels-9sites_20140922-pAKT2-
20140917\results-Cy5.csv
%
% To extend the code to different selected data or a different text file,
% generate a function instead of a script.

% Auto-generated by MATLAB on 2014/10/10 12:18:18

%% Initialize variables.
filename = strcat(mypath,'\results-Cy5.csv');
delimiter = ',';
startRow = 2;

%% Format string for each line of text:
%   column1: double (%f)
%      column2: text (%s)
%   column3: double (%f)
%      column4: double (%f)
%   column5: double (%f)
%      column6: double (%f)
%   column7: double (%f)
%      column8: double (%f)
%   column9: double (%f)
%      column10: double (%f)
```

```
%   column11: double (%f)
%      column12: double (%f)
%   column13: double (%f)
%      column14: double (%f)
%   column15: double (%f)
%      column16: double (%f)
%   column17: double (%f)
%      column18: double (%f)
%   column19: double (%f)
%      column20: double (%f)
%   column21: double (%f)
% For more information, see the TEXTSCAN documentation.
formatSpec = '%f%s%f%f%f%f%f%f%f%f%f%f%f%f%f%f%f%f%f%f%f%[^\n\r]';

%% Open the text file.
fileID = fopen(filename,'r');

%% Read columns of data according to format string.
% This call is based on the structure of the file used to generate this
% code. If an error occurs for a different file, try regenerating the code
% from the Import Tool.
dataArray = textscan(fileID, formatSpec, 'Delimiter', delimiter, 'EmptyValue'
,NaN,'HeaderLines' ,startRow-1, 'ReturnOnError', false);

%% Close the text file.
fclose(fileID);

%% Post processing for unimportable data.
% No unimportable data rules were applied during the import, so no post
% processing code is included. To generate code which works for
% unimportable data, select unimportable cells in a file and regenerate
% theCy5
% script.

%% Create output variable

resultsCy5 = table(dataArray{1:end-1}, 'VariableNames',
{'VarName1','Well','Area','Mean','StdDev','Min','Max','X','Y','Perim','Circ','IntDen','Median
','Skew','Kurt','VarName16','RawIntDen','Slice','AR','Round','Solidity'});
%% Clear temporary variables
resultsCy5.Well = cellfun(@(x) x(length(x)-26:length(x)-
21),resultsCy5.Well,'UniformOutput',false);
clearvars filename delimiter startRow formatSpec fileID dataArray ans;
```

Data processing (Matlab)

```
%% Cytell Well Mean & IQR Summary
%
% read Cytell data table and convert to matrix
% plot well Mean color matrices
% 2014/10/10 Jerry Lin
%

%% Read datatables

tempcell=varfun(@mean,resultsDAPI,'InputVariables','Mean','Groupingvariables','Well');
WellLabel=reshape(tempcell.Well,10,6)';
WellMeanDAPI=reshape(tempcell.mean_Mean,10,6)';
```

```
tempcell=varfun(@mean,resultsFITC,'InputVariables','Mean','Groupingvariables','Well');
WellMeanFITC=reshape(tempcell.mean_Mean,10,6)';

tempcell=varfun(@mean,resultsCy3,'InputVariables','Mean','Groupingvariables','Well');
WellMeanCy3=reshape(tempcell.mean_Mean,10,6)';

tempcell=varfun(@mean,resultsCy5,'InputVariables','Mean','Groupingvariables','Well');
WellMeanCy5=reshape(tempcell.mean_Mean,10,6)';


%% plotting
figure;
x = repmat(1:10,6,1);
y = repmat((1:6)',1,10);

subplot(2,2,1);
imagesc(WellMeanDAPI);
title('DAPI','FontSize',14);
colorbar;

subplot(2,2,2);
imagesc(WellMeanFITC);
title('FITC','FontSize',14);
colorbar;

subplot(2,2,3);
imagesc(WellMeanCy3);
title('Cy3','FontSize',14);
colorbar;

subplot(2,2,4);
imagesc(WellMeanCy5);
title('Cy5','FontSize',14);
colorbar;
return;
```
